# Belinostat for Relapsed or Refractory Peripheral T-Cell Lymphoma

**DOI:** 10.6004/jadpro.2016.7.2.6

**Published:** 2016-03-01

**Authors:** Katelyn Hood, Arpita Shah

**Affiliations:** Nationwide Children’s Hospital, Columbus, Ohio, and Georgia Regents Medical Center, The University of Georgia College of Pharmacy, Augusta, Georgia

**Belinostat for Relapsed or Refractory Peripheral T-Cell Lymphoma**

A continuing education article for nurse practitioners, physician assistants, clinical nurse specialists, advanced degree nurses, oncology and hematology nurses, pharmacists, and physicians.

**Release date:** March 15, 2016

**Expiration date:** March 15, 2017

**Expected time to complete this activity as designed:** 0.75 hour

**Meniscus Educational Institute**

3131 Princeton Pike,

Building 1, Suite 205A

Lawrenceville, NJ 08648

Voice: 609-246-5000

Fax: 609-449-7969

E-mail: lrubin@meniscusedu.com

**Journal of the Advanced Practitioner in Oncology**

94 N. Woodhull Road

Huntington, NY 11743

Voice: 631-692-0800

Fax: 631-692-0805

E-mail: claudine@harborsidepress.com

© *2016, Meniscus Educational Institute. All rights reserved.*

## Faculty

**Katelyn Hood, PharmD,** Nationwide Children’s Hospital, Columbus, Ohio

**Arpita Shah, PharmD,** Georgia Regents Medical Center, The University of Georgia College of Pharmacy, Augusta, Georgia

## Activity Rationale and Purpose

Peripheral T-cell lymphomas (PTCLs) represent an uncommon heterogenous group of neoplasms that make up approximately 10% to 15% of non-Hodgkin lymphomas. PTCL, not otherwise specified (NOS) is the most common form, whose incidence in the United States has been increasing, possibly due to better diagnostic methods. PTCLs represent a rare and aggressive subgroup of NHLs that do not respond favorably to traditional chemotherapies. Since the majority of patients with PTCL experience disease relapse or disease that is refractory to previous agents, the continued development of novel targeted therapies is critical and necessary in order to improve outcomes in this aggressive, difficult to treat, heterogeneous group of malignant disorders.

The FDA approval of belinostat provides advanced practitioners with an additional option to offer heavily pre-treated patients with relapsed/refractory PTCL who did not achieve a desirable response to traditional chemotherapy agents. Belinostat is a favorable treatment option for these patients because of its manageable toxicity profile and its ability to be used in patients with baseline thrombocytopenia. The safety and efficacy of belinostat is currently being evaluated for use in combination with traditional front-line therapies for the treatment of PTCL. The results of these trials have the potential to expand belinostat’s place in therapy and challenge the traditional treatment approach for PTCL.

## Intended Audience

The activity’s target audience will consist of nurse practitioners, physician assistants, clinical nurse specialists, advanced degree nurses, oncology and hematology nurses, pharmacists, and physicians.

## Learning Objectives

- Describe the mechanism of action of belinostat
- Identify situations in which adverse effects would require dose modification or discontinuation of belinostat
- Discuss the recommended administration and treatment schedule for belinostat

## Continuing Education

**Statement of Credit—Participants who successfully complete this activity (including the submission of the post-test and evaluation form) will receive a statement of credit.**

**Physicians.** The Meniscus Educational Institute is accredited by the Accreditation Council for Continuing Medical Education (ACCME) to provide continuing medical education for physicians.

The Meniscus Educational Institute designates this journal article for a maximum of 0.75 *AMA PRA Category 1 Credits*™. Physicians should claim only the credit commensurate with the extent of their participation in the activity.

**Nurses.** This activity for 0.75 contact hour is provided by the Meniscus Educational Institute.

The Meniscus Educational Institute is accredited as a provider of continuing nursing education by the American Nurses Credentialing Center’s Commission on Accreditation.

**Pharmacists.** The knowledge-based accredited education journal articles are intended for pharmacists involved in the care of cancer patients. This educational activity is sponsored by the Meniscus Educational Institute.

The Meniscus Educational Institute is accredited by the Accreditation Council for Pharmacy Education (ACPE) as a provider of continuing pharmacy education. The ACPE Universal Activity Number assigned to this program, for 0.75 contact hour, is 0429-9999-16-003-H01-P.

## Financial Disclosures

All individuals in positions to control the content of this program (eg, planners, faculty, content reviewers) are expected to disclose all financial relationships with commercial interests that may have a direct bearing on the subject matter of this continuing education activity. Meniscus Educational Institute has identified and resolved all conflicts of interest in accordance with the MEI policies and procedures. Participants have the responsibility to assess the impact (if any) of the disclosed information on the educational value of the activity.

**Faculty**

**Katelyn Hood, PharmD,** has nothing to disclose.

**Arpita Shah, PharmD,** has nothing to disclose.

**Lead Nurse Planner**

**Wendy J. Smith, ACNP, AOCN®,** has nothing to disclose.

**Planners**

**Jeannine Coronna** has nothing to disclose.

**Claudine Kiffer** has nothing to disclose.

**Terry Logan, CHCP,** has nothing to disclose.

**Pamela Hallquist Viale, RN, MS, CNS, ANP,** has nothing to disclose.

**Lynn Rubin** has nothing to disclose.

**Content Reviewers**

**Glenn Bingle, MD, PhD, FACP,** has nothing to disclose.

**Kate D. Jeffers, PharmD, BCOP,** has nothing to disclose.

**Karen Abbas, MS, RN, AOCN®,** has nothing to disclose.

**Wendy J. Smith, ACNP, AOCN®,** has nothing to disclose.

## Disclaimer

This activity has been designed to provide continuing education that is focused on specific objectives. In selecting educational activities, clinicians should pay special attention to the relevance of those objectives and the application to their particular needs. The intent of all Meniscus Educational Institute educational opportunities is to provide learning that will improve patient care. Clinicians are encouraged to reflect on this activity and its applicability to their own patient population.

The opinions expressed in this activity are those of the faculty and reviewers and do not represent an endorsement by Meniscus Educational Institute of any specific therapeutics or approaches to diagnosis or patient management.

## Product Disclosure

This educational activity may contain discussion of published as well as investigational uses of agents that are not approved by the US Food and Drug Administration. For additional information about approved uses, including approved indications, contraindications, and warnings, please refer to the prescribing information for each product.

## How to Earn Credit

To access the learning assessment and evaluation form online, visit www.meniscusce.com

**Statement of Credit:** Participants who successfully complete this activity (including scoring of a minimum of 70% on the learning assessment and complete and submit the evaluation form with an E-mail address) will be able to download a statement of credit.

## ARTICLE

Non-Hodgkin lymphomas (NHLs) are a diverse group of lymphoproliferative disorders that affect B cells, T cells, and natural killer (NK) cells (Savage, 2007). Approximately 71,850 individuals in the United States were diagnosed with NHL in 2015, with an estimated number of deaths approaching 20,000 (National Cancer Institute, 2014). Peripheral T-cell lymphomas (PTCLs) are a subgroup of rare and aggressive NHLs that derive from malignant proliferation of mature T lymphocytes/NK cells and account for approximately 10% to 15% of all NHLs (Skarbnik, Burki & Pro, 2013; Zheng et al., 2014).

The PTCLs are divided into multiple subtypes including but not limited to PTCL–not otherwise specified (PTCL-NOS; 26%), angioimmunoblastic T-cell lymphoma (AITL; 18.5%), NK/T-cell lymphoma (10%), adult T-cell leukemia/lymphoma (ATLL; 10%), anaplastic large cell lymphoma (*ALK*)-positive lymphoma (ALCL; 7%), and *ALK*-negative ALCL (6%; Vose, Armitage, & Weisenburger, 2008).

The PTCLs usually affect patients ≥ 60 years of age, with the majority (68%) initially presenting with disseminated disease (Gallamini et al., 2004). When compared with their B-cell NHL counterparts, most PTCL histologies, with the exception of *ALK*-positive ALCL, are more difficult to treat and are associated with worse progression-free survival (PFS) and overall survival (OS; Skarbnik et al., 2013).

Traditionally, treatment algorithms for PTCL have been extrapolated from regimens used to treat B-cell lymphomas and usually consist of an anthracycline-based chemotherapy regimen such as cyclophosphamide, doxorubicin, vincristine, and prednisone (CHOP); CHOP plus etoposide (CHOEP); etoposide, prednisone, vincristine, cyclophosphamide, and doxorubicin (EPOCH); or cyclophosphamide, vincristine, doxorubicin, and dexamethasone (hyper-CVAD; National Comprehensive Cancer Network [NCCN], 2015; Leukemia & Lymphoma Society, 2014). Although conventional chemotherapy regimens are utilized as front-line therapy, they are associated with a 5-year OS of 14% to 35% for the most common PTCL histologies, excluding ALK-positive ALCL, which is traditionally associated with a more favorable prognosis (5-yr OS of 70% to 79%; Poole, 2014; Sharbnik et al., 2013). Because of a lack of robust, prospective clinical data evaluating different therapeutic strategies, enrollment in a clinical trial is recommended as first-line management of most PTCL histologies (Skarbnik et al., 2013; Zain & O’Connor, 2010).

Given the poor outcomes associated with conventional chemotherapies as front-line therapy, the roles of high-dose chemotherapy followed by autologous stem cell rescue (HDT/ASCR) and allogeneic stem cell transplant (allo-SCT) have been investigated as consolidation therapies. However, SCT is not a viable treatment option for older or unfit patients or for those who do not achieve a significant response to front-line systemic therapies (Skarbnik et al., 2013). As a result, first-line allogeneic or autologous SCT is effective only in the younger patient population in terms of clinical response (Bodiford, Bodge, Talbott, & Reddy, 2014). Unfortunately, the majority of patients with PTCL will experience disease relapse and/or disease that is refractory to previous agents, including those who have undergone SCT.

Treatment options for relapsed or refractory PTCL include alemtuzumab (Campath), bortezomib (Velcade), gemcitabine, dose-adjusted EPOCH, or novel agents such as pralatrexate (Folotyn) and romidepsin (Istodax)—the first histone deacetylase (HDAC) inhibitor (class I selective) approved for the treatment of PTCL (Celgene, 2014; NCCN, 2015). These agents are associated with an overall response rates (ORR) and a median duration of response (DOR) of 25% to 60% and 3.5 to 17 months, respectively (Coiffier et al., 2012; Enblad et al., 2004; O’Connor et al., 2011; Sallah, Wan, & Nguyen, 2001; Zinzani et al., 1998, 2005, 2010). Belinostat (Beleodaq) is also an HDAC inhibitor that was granted an accelerated approval by the US Food and Drug Administration (FDA) in July 2014 as monotherapy for the treatment of relapsed or refractory PTCL (FDA, 2014). Romidepsin and belinostat are the only HDAC inhibitors approved for the treatment of PTCL.

## MECHANISM OF ACTION

Inhibition of HDAC induces histone acetylation, leading to increased expression of tumor-suppressor genes and accumulation of acetylated histone proteins. This accumulation ultimately causes disruption of cell-cycle progression, inhibition of angiogenesis, and apoptosis (Figure; Bodiford et al., 2014; Marks, Richon, & Rifkind, 2000; Poole, 2014; Steele et al., 2008; Emanuele, Lauricella, & Tesoriere, 2008).

**Figure 1:**
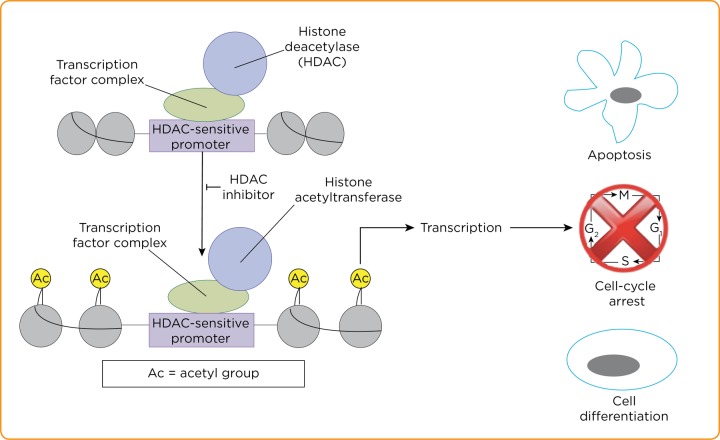
HDAC inhibition: Mechanism of action. Adapted from Bodiford, Bodge, Talbott, & Reddy (2014).

Approximately 18 different HDACs have been identified and categorized into four classes, with classes I and II HDACs being the major targets for HDAC inhibition. Classes I and II HDACs are responsible for regulating cell proliferation and suppressing apoptosis in malignant cells, respectively (Bodiford et al., 2014). In a study evaluating the expression of HDAC1, HDAC2, HDAC6, and acetylated histone 4 (H4) in patients with lymphoma, HDAC1 was found to be overexpressed to a larger degree in patients with PTCL compared with patients with diffuse large B-cell lymphoma (Marquard et al., 2009).

In vitro, belinostat exhibits pan-HDAC inhibition (i.e., classes I, II, and IV HDAC inhibition) and potent growth inhibitory and proapoptotic activities in a variety of tumor cells, including PTCL cells, at nanomolar concentrations (Marks et al., 2000; Poole, 2014; O’Connor, 2015; Steele et al., 2008). At this time, it is unclear which class of HDACs, when inhibited, actually account for the activity of belinostat in PTCL. Even though the importance of classes II and IV HDAC inhibition is not completely understood, pan-HDAC inhibition with belinostat, as compared with class I selective HDAC inhibition with romidepsin, is presumed to be advantageous (Khan et al., 2008; O’Connor et al, 2015). Additionally, available studies have not demonstrated increased toxicity with pan-HDAC inhibition (Khan et al., 2008).

## PHARMACOKINETICS AND PHARMACODYNAMICS

Belinostat demonstrates three-compartment pharmacokinetics, with an elimination half-life ranging from 0.3 to 1.3 hours (Steele et al., 2008). The volume of distribution approaches total body water (~114 L/m2), and the majority of the drug (93%–96%) is protein bound, indicating limited tissue distribution (Poole, 2014). Belinostat is primarily metabolized by the hepatic enzyme UGT1A1 (80%–90%) and to a lesser extent by CYP2A6, CYP2C9, and CYP3A4 (Poole, 2014; Wang et al., 2013). Strong inhibitors of UGT1A1, such as atazanavir (Reyataz), are expected to increase exposure to belinostat; therefore, this combination should be avoided. Elimination of belinostat occurs predominantly through metabolism, with < 2% of the dose recovered as unchanged drug in the urine (Steele et al., 2008).

## PHARMACOGENOMICS

Approximately 20% of African Americans, 10% of Caucasians, and 2% of Asians are homozygous for the UGT1A1*28 allele, which is a genetic polymorphism that results in reduced enzyme activity (Spectrum Pharmaceuticals, Inc., 2014). Since belinostat is primarily metabolized by UGT1A1, patients who are homozygous for the UGT1A1*28 allele could experience decreased belinostat clearance and increased toxicities. Therefore, patients with a known polymorphism should receive reduced doses of belinostat to minimize the dose-limiting toxicities (Spectrum Pharmaceuticals, Inc., 2014). At this time, routine genetic testing for the UGT1A1*28 allele is not required before belinostat initiation. However, further studies are warranted to determine the true impact of this polymorphism on the safety profile of belinostat (Wang et al., 2013).

## CLINICAL STUDIES

Belinostat has demonstrated antitumor activity in vitro and in vivo against a variety of tumor cell lines at nanomolar concentrations (Plumb et al., 2003). In a phase I dose-finding study involving 46 patients with advanced solid tumors, belinostat was found to have a manageable adverse event (AE) profile at the maximum tolerated dose of 1,000 mg/m² administered intravenously on days 1 to 5 of a 21-day cycle. About 50% of the patients received the maximum tolerated dose, and disease stabilization was observed in 39% of the patients (Steele et al., 2008).

In a phase II, open-label trial, belinostat was evaluated in heavily pretreated (1 to 12 prior regimens) patients with relapsed or refractory PTCL (n = 24) or cutaneous T-cell lymphoma. Approximately 21% of patients with PTCL had received autologous SCT, and 50% had stage IV disease. Belinostat was administered as a 30-minute IV infusion at 1,000 mg/m² on days 1 to 5 of a 21-day cycle, and therapy was continued for up to eight cycles. Patients received a median of two cycles of belinostat (range: 1–9). The ORR was 25%, with complete response (CR) and partial response (PR) rates of 8.3% and 16.7%, respectively. The median time to disease progression was 82 days, and the median DOR was 109 days (Foss et al., 2015).

In the phase II BELIEF trial, the efficacy and safety of single-agent belinostat were evaluated in 129 heavily pretreated patients with relapsed or refractory PTCL who had received a median of two prior therapies. Patients were included in this study if they had failed at least one prior therapy, had received no prior therapy with an HDAC inhibitor, and had a platelet count ≥ 50 × 10^9^/L. The median age of enrolled patients was 64 years old, and 52% were male. The majority of patients (97%) received CHOP or a CHOP-like regimen in the past, with 21% of the patients having undergone SCT prior to enrollment. Belinostat was administered as a 30-minute intravenous infusion at 1,000 mg/m² on days 1 to 5 of a 3-week cycle, and therapy was continued until disease progression or unacceptable toxicity occurred.

Of the 120 patients eligible for efficacy evaluation, the ORR was 26%, with CR and PR rates of 11% and 15%, respectively. In patients with the two most common subtypes of PTCL (PTCL-NOS and AITL), the median ORR was 23.3% and 45.5%, respectively. The median time to response was 5.6 weeks, and the median DOR was 13.6 months. Results of this trial led to belinostat’s FDA approval for the treatment of relapsed or refractory PTCL (O’Connor et al., 2015).

## ADVERSE EFFECTS

Among patients receiving belinostat, the most common AEs (reported in > 25%) were nausea, fatigue, pyrexia, anemia, and vomiting (Table 1). The most common grade 3/4 AEs (≥ 5%) were anemia (11%), thrombocytopenia (7%), dyspnea (6%), neutropenia (6%), fatigue (5%), and pneumonia (5%). The most common serious AEs (> 2%) were anemia, increased serum creatinine, infection, multiorgan failure, pneumonia, pyrexia, and thrombocytopenia (O’Connor et al., 2015).

**Table 1:**
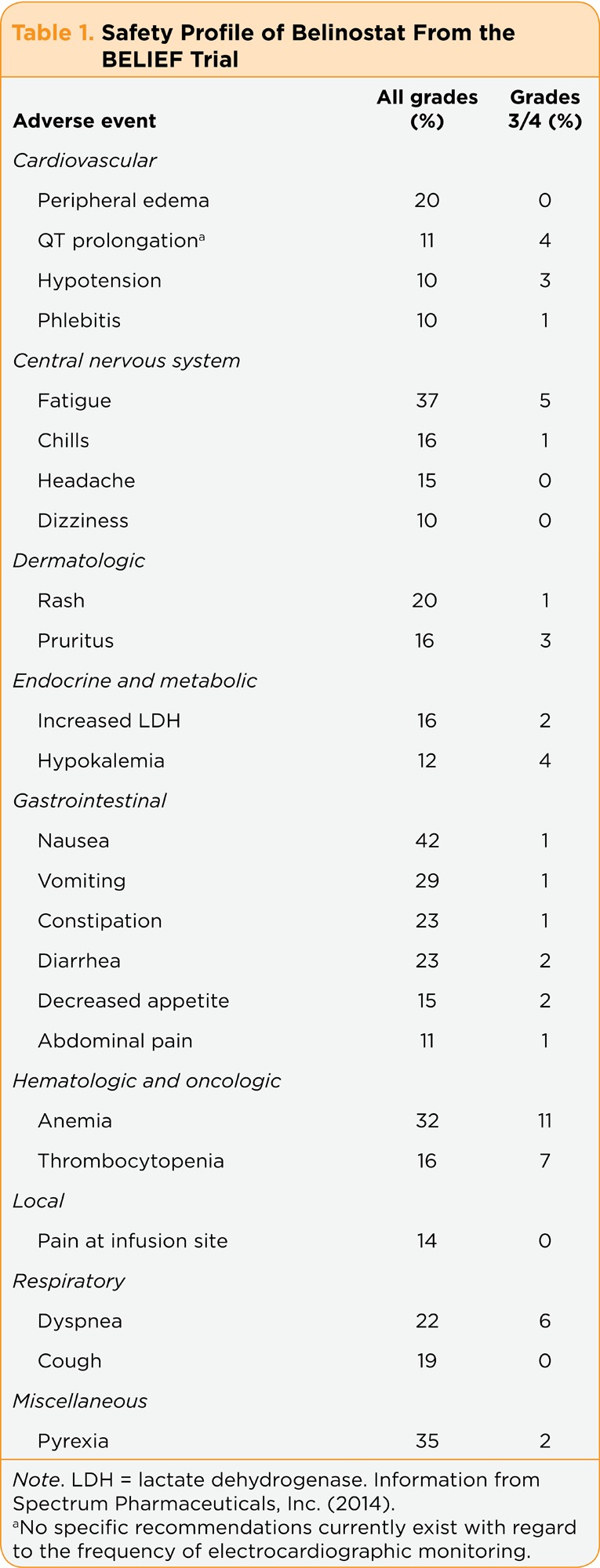
Safety Profile of Belinostat From the BELIEF Trial

In the BELIEF trial, patients with a platelet count of between 50 and 100 × 10^9^/L were able to tolerate belinostat with 98% dose intensity; hence, belinostat may be a worthwhile treatment option in patients with baseline thrombocytopenia. Approximately 19.4% of patients who received belinostat discontinued therapy due to AEs, and 12.4% of patients required a dose adjustment due to AEs. The most frequent causes of drug discontinuation due to AEs included anemia, febrile neutropenia, fatigue, and multiorgan failure. Overall, belinostat appears to be well tolerated, with a low incidence of bone marrow suppression—a unique characteristic when compared with other agents available for the management of relapsed or refractory PTCL, including romidepsin (O’Connor et al., 2015).

## DOSING AND ADMINISTRATION

The recommended dose of belinostat is 1,000 mg/m² administered as an intravenous infusion over 30 minutes once daily on days 1 to 5 of a 21-day cycle. Therapy should be continued until disease progression or occurrence of unacceptable AEs. The absolute neutrophil count (ANC) should be ≥ 1.0 × 10^9^/L, and the platelet count should be ≥ 50 × 10^9^/L prior to the start of each cycle and prior to resuming treatment following toxicity.

Dose reduction is recommended in patients experiencing hematologic toxicities, grade 3/4 nonhematologic toxicities, or reduced UGT1A1 activity (Table 2). Belinostat should be discontinued in patients who have required two dose reductions and still have recurrent ANC nadirs < 0.5 × 10^9^/L, recurrent platelet count nadirs < 25 × 10^9^/L, or recurrent grade 3 or 4 toxicity. Due to the risk of toxicity, complete blood cell counts should be monitored at baseline and weekly throughout treatment (O’Connor et al., 2015; Spectrum Pharmaceuticals, Inc., 2014).

**Table 2:**
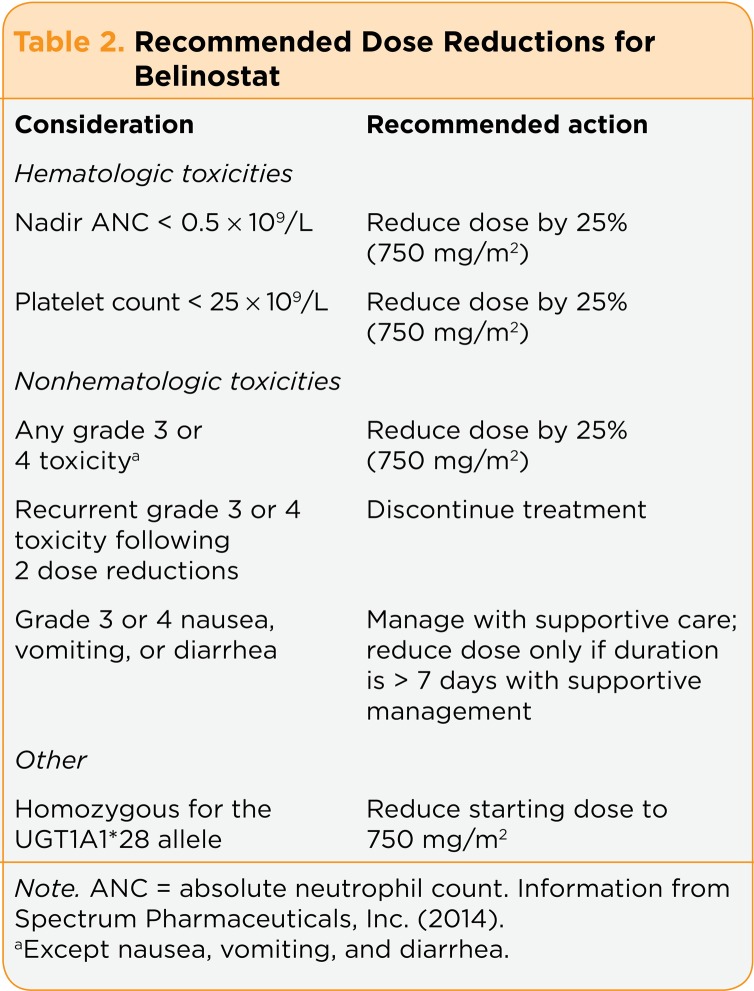
Recommended Dose Reductions for Belinostat

Since belinostat is metabolized by the liver, hepatic impairment is expected to increase belinostat exposure. As patients with moderate to severe hepatic impairment were excluded from clinical trials, a dose cannot be recommended for these patients. No dose adjustments are required in patients with creatinine clearance (CrCl) > 39 mL/min; however, there are insufficient data to recommend a dose of belinostat in patients with a CrCl ≤ 39 mL/min, since patients with renal insufficiency have been excluded from clinical studies (O’Connor et al., 2015; Spectrum Pharmaceuticals, Inc., 2014).

Belinostat is available as a 500-mg single-use vial of lyophilized powder for reconstitution. The vial should be reconstituted with 9 mL of sterile water for injection, creating a final concentration of 50 mg/mL. The reconstituted vial can be stored at room temperature (15°C–25°C; 59°F–77°F) for up to 12 hours. Prior to administration, belinostat should be further diluted in a 250-mL bag of 0.9% sodium chloride for injection; the infusion bag can be stored for up to 36 hours (including infusion time) at room temperature. Belinostat should be filtered using a 0.22-µm inline filter for administration. The infusion can be extended to 45 minutes if infusion-site pain or other infusion-related signs/symptoms occur (Spectrum Pharmaceuticals, Inc., 2014).

## IMPLICATIONS FOR THE FUTURE

The NCCN has included belinostat in its guidelines as a potential second-line agent for the treatment of relapsed or refractory PTCL. However, no one agent is preferred over another as second-line therapy for relapsed or refractory PTCL. The choice of therapy should be individualized and guided based on PTCL subtype, toxicity profile, and dosing schedule (NCCN, 2015).

As a condition of the accelerated approval of belinostat, the FDA is requiring two additional clinical trials to be conducted in patients with PTCL: a phase I dose-finding study of belinostat in combination with CHOP (BelCHOP; NCT01839097) followed by a phase III study comparing the efficacy of BelCHOP vs. CHOP alone as front-line therapy (FDA, 2014). Since CHOP is often utilized as a first-line regimen for the treatment of PTCL, the results of these trials will be crucial in potentially expanding belinostat’s role as monotherapy or in combination with other agents in the front-line setting.

## SUMMARY

The PTCLs represent a rare and aggressive subgroup of NHLs that do not respond favorably to traditional chemotherapies. Since the majority of patients with PTCL experience disease relapse or disease that is refractory to previous agents, the continued development of novel targeted therapies is critical and necessary to improve outcomes in this aggressive, difficult-to-treat, heterogeneous group of malignant disorders.

The FDA approval of belinostat provides clinicians with an additional option to offer heavily pretreated patients with relapsed or refractory PTCL who did not achieve a desirable response to traditional chemotherapy agents. Belinostat may be a worthwhile treatment option for these patients because of its manageable toxicity profile and its ability to be used in patients with baseline thrombocytopenia (platelet count 50–100 × 10^9^/L). The safety and efficacy of belinostat are currently being evaluated for use in combination with traditional front-line therapies for the treatment of PTCL. The results of these trials have the potential to expand belinostat’s place in therapy and challenge the traditional treatment approach for PTCL.
